# Vitamin D Status and SARS-CoV-2 Infection in a Cohort of Kidney Transplanted Patients

**DOI:** 10.3390/nu14020317

**Published:** 2022-01-13

**Authors:** Anna Regalia, Matteo Benedetti, Silvia Malvica, Carlo Alfieri, Mariarosaria Campise, Donata Cresseri, Maria Teresa Gandolfo, Federica Tripodi, Giuseppe Castellano, Piergiorgio Messa

**Affiliations:** 1 Department of Nephrology, Dialysis, and Renal Transplantation, Fondazione IRCCS Ca’ Granda Ospedale Maggiore Policlinico, 20122 Milan, Italy; matteo.benedetti@unimi.it (M.B.); silvia.malvica@unimi.it (S.M.); carlo.alfieri1@gmail.com (C.A.); maria.campise@policlinico.mi.it (M.C.); donata.cresseri@policlinico.mi.it (D.C.); mariateresa.gandolfo@policlinico.mi.it (M.T.G.); fedetr89@gmail.com (F.T.); giuseppe.castellano@unimi.it (G.C.); piergiorgio.messa@gmail.com (P.M.); 2 Department of Clinical Sciences and Community Health, University of Milan, 20122 Milano, Italy

**Keywords:** vitamin D, SARS-CoV-2 infection, kidney transplantation

## Abstract

Background: Recently the protective role of 25-hydroxyvitamin D (25(OH)D) against viral infections has been hypothesized. We evaluated the association between vitamin D status and SARS-CoV-2 infection susceptibility and severity in a cohort of kidney transplanted patients (KTxp). Methods: A total of 61 KTxp with SARS-CoV-2 infection (COV+) were matched with 122 healthy KTxp controls (COV−). Main biochemical parameters at 1, 6, and 12 months before SARS-CoV-2 infection were recorded. Vitamin D status was considered as the mean of two 25(OH)D measures obtained 6 ± 2 months apart during the last year. The severity of SARS-CoV-2 infection was based on the need for hospitalization (HOSP+) and death (D+). Results: 25(OH)D levels were lower in COV+ than in controls [19(12–26) vs. 23(17–31) ng/mL, *p* = 0.01]. No differences among the other biochemical parameters were found. The SARS-CoV-2 infection discriminative power of 25(OH)D was evaluated by ROC-curve (AUC 0.61, 95% CI 0.5–0.7, *p* = 0.01). 25(OH)D was not significantly different between HOSP+ and HOSP− [17(8–25) vs. 20(15–26) ng/mL, *p* = 0.19] and between D+ and D− [14(6–23) vs. 20(14–26) ng/mL, *p* = 0.22] and had no significant correlation with disease length. Conclusions: During the year preceding the infection, 25(OH)D levels were lower in COV+ KTxp in comparison with controls matched for demographic features and comorbidities. No significant association between vitamin D status and SARS-CoV-2 infection related outcomes was found.

## 1. Introduction

The 2019 coronavirus disease (COVID-19), a potentially life-threatening respiratory and systemic disorder caused by severe acute respiratory syndrome coronavirus 2 (SARS-CoV-2) has emerged as a major global concern since 2020 [1], for which no proven therapy has been identified so far.

Since immunomodulatory and anti-inflammatory properties have been hypothesized for vitamin D [2,3] a rising interest of the scientific community was directed towards a potential preventive and therapeutic role of vitamin D supplementation in SARS-CoV-2 infected patients. On the other side, vitamin D deficiency has been associated with higher frequency and severity of SARS-CoV-2 infection in many observational studies, both in the general population and in patients suffering from comorbid conditions that can impair the global immune response, such as diabetes and cardiovascular disease [4]. Patients with chronic kidney disease (CKD) and kidney transplantation (KTx) have high rates of vitamin D deficiency, for several reasons, such as steroid therapy and increasing age at transplantation [5,6]. Furthermore, KTx patients (KTxp) are at high risk of SARS-CoV-2 infection incidence and severity [7,8].

To the best of our knowledge, there are no published studies specifically addressing the association between 25(OH)D and SARS-CoV-2 infection in this particular subgroup of patients.

We conducted a retrospective monocentric study to evaluate the possible role of vitamin D status for risk and severity of developing SARS-CoV-2 infection in a cohort of KTx patients followed-up in our center.

## 2. Material and Methods

### 2.1. Studied Cohort and Design

The study was carried out among the KTxp followed in our Nephrology Department (Fondazione IRCCS Ca’ Granda Ospedale Maggiore Policlinico of Milan, Italy). Between the 1 March 2020 and the 21 April 2021, 82 out of 1558 KTxp were diagnosed with SARS-CoV-2 infection (COV+).

The criteria for the suspicion of SARS-CoV-2 infection were: the possible contact with another person in whom SARS-CoV-2 infection was confirmed or the presence of one or more symptoms compatible with COVID-19 (fever, respiratory symptoms, diarrhea, taste, and smell dysfunction). Diagnosis was always confirmed by the positivity of the RNA test via nasopharyngeal swab.

Sixty-one COV+ KTxp and 122 KTxp without SARS-CoV-2 infection (COV−) were included in the study. They were matched 1:2 for sex, age, KTx vintage, presence of treated diabetes, hypertension (requiring at least one anti-hypertensive medication) and symptomatic heart disease (NYHA II or higher).

The main criterion of inclusion was the availability of at least two measurements of 25(OH)D during the last 12 months, at least 6 ± 2 months apart. For COV+ the baseline was the day of the first SARS-CoV-2 positivity, whereas for COV− the baseline was the same of the corresponding COV+ patient.

Twenty-one COV+ KTxp were excluded due to the unavailability of vitamin D status parameters in the year before the infection.

Body mass index (BMI), serum creatinine, daily urinary protein excretion, mineral metabolism (MM) parameters (serum calcium, phosphorus, magnesium, intact parathormone, 1,25-dihydroxyvitamin D) serum albumin and C-reactive protein (CRP), were also collected.

For statistical analyses, each parameter was considered as the mean of the collected values in the last year.

Immunosuppressive medications, therapy with renin-angiotensin-aldosterone system inhibitors (iRAAS), and vitamin D supplementation (considered as cholecalciferol or calcidiol intake for at least three months in the last 12 months) were also recorded.

**Figure 1:**
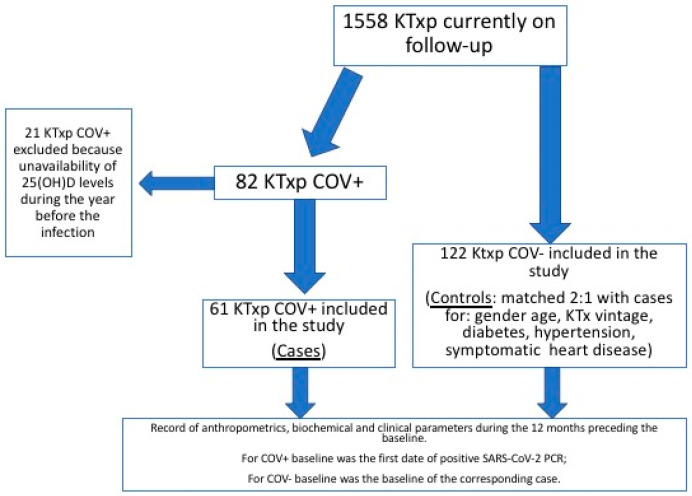
Flow-chart of the study. KTx, kidney transplantation; KTxp, kidney transplanted patients; COV+: KTxp with SARS-CoV-2 infection; COV-, KTxp without SARS-CoV-2 infection; 25(OH)D, 25-hydroxyvitamin D.

In Figure 1, the diagram of the study is shown.

The severity of SARS-CoV-2 infection was evaluated by means of SARS-CoV-2 related outcomes: the need for hospitalization (HOSP+) and death (D+).

### 2.2. Laboratory Measures

#### 2.2.1. SARS-CoV-2 Assessment

SARS-CoV-2 positivity was assessed by positivity of the RNA test via nasopharyngeal swab according to international guidelines [9].

#### 2.2.2. Vitamin D

Levels of 25-hydroxyvitamin D have been assessed in serum specimens by enzyme-immunoassay (Kit EIA AC-57FI-immunodiagnostic system Boldon, UK), using a highly specific sheep 25-hydroxyvitamin D antibody and enzyme (horseradish peroxidase) labelled avidin. The sensitivity threshold was 5 nmol/mL (2 ng/mL). The specificity of the antiserum was assessed with the following analytes at 50% binding of the zero calibrator; cross reactivity: 25-hydroxyvitamin D3 100%; 25-hydroxyvitamin D2 75%; 24-hydroxyvitamin D3 100%; cholecalciferol (D3) < 0.01%; ergocalciferol (D2) < 0.30%. The intra-assay precision was calculated from 10 duplicate determinations of two samples each, performed in a single assay (CV between 5.3 and 6.7%). The inter-assay precision was calculated from duplicate determinations of two samples performed in eleven assays (CV between 4.6 and 8.7%). This method has been demonstrated to give results strictly related to the ones obtained by two different RIA methods, which are considered to be the gold-standard for 25-hydroxyvitamin D assessment (EIA method vs. method 1 RIA and vs. method 2 RIA (http://www.idsplc.com/int/products/25-hydroxy-vitamin-d-eia-ac-57f1#description (accessed on 10 December 2021)).

All of the other biochemical parameters were evaluated according to routine methodology used in our central laboratory.

### 2.3. Statistical Analyses

Continuous variables were expressed as median value and interquartile range (25th–75th percentile) and categorical variables were reported as number of cases (n) and relative percentage. Differences among groups were determined by Kruskal–Wallis test and differences among percentages were determined by χ^2^ test or Fisher’s exact test if appropriate. Linear regression was used to evaluate the association between continuous variables.

The discriminative power of vitamin D status on risk of SARS-CoV-2 infection was analyzed by means of Receiver Operating Characteristic (ROC) curve. In all statistical analyses significance was set for *p* values < 0.05.

Statistical analysis was performed using the software Medcalc^®^ and Statistica^®^ version 10.

## 3. Results

### 3.1. Overall Cohort Characteristics

Table 1 reports the main features of the studied cohort, considered as a whole (overall cohort) and divided into COV+ and COV−.

In the overall cohort (n. 183 patients), the median age was 53 years (range 45–64) and the median transplant vintage was 7.6 years (2.6–15.3). One-hundred eleven patients were male (60.7%). The prevalence of diabetes, hypertension, and symptomatic heart disease was 18%, 85%, and 3%, respectively.

As expected, considering the design of the study, there were no statistically significant differences between COV+ and COV− in these general characteristics.

### 3.2. Vitamin D Status and Other Biochemical Parameters

Table 2 reports the differences in 25(OH)D levels and other biochemical parameters in 12 months before baseline.

Vitamin D status was significantly worse in COV+ than in COV−, with median 25(OH)D levels of 19(12–26) ng/mL vs. 23(17–31) ng/mL (*p* = 0.01).

No differences between COV+ and COV− were found in the other MM parameters.

Median serum creatinine was 1.4 mg/dl (range 1.2–1.8), with mild or absent proteinuria, without differences between COV+ and COV−.

Albumin and C-reactive protein values were normal in the majority of patients, with no differences between the two groups.

### 3.3. Discriminative Role of Vitamin D Status on COV Positivity

**Figure 2:**
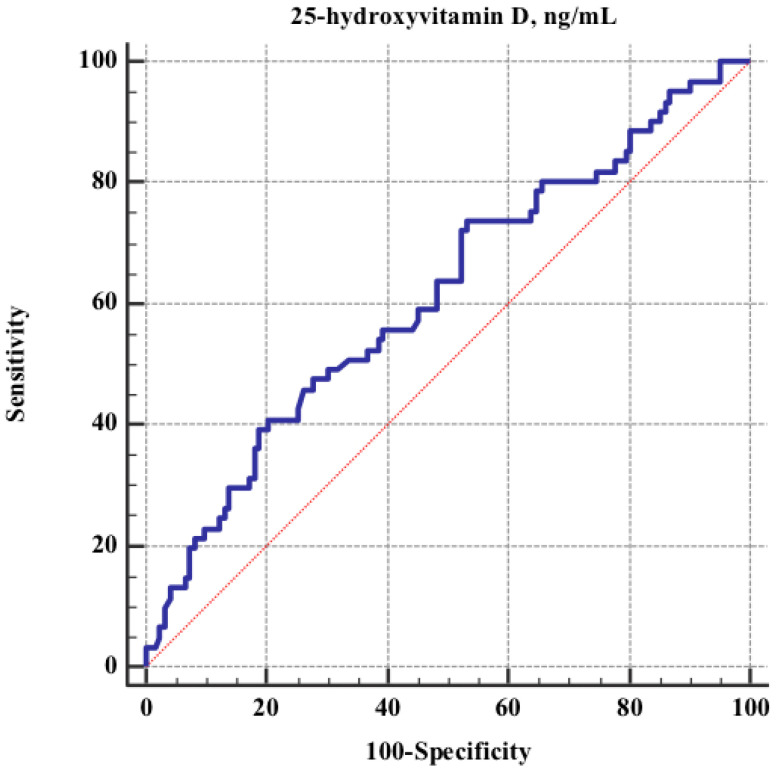
ROC (Receiver Operating Characteristic) curve for discriminative power of vitamin D status respect COV positivity. AUC 0.61 (95% CI 0.6–0.7), *p* = 0.01; Youden Index 0.21; criterion value corresponding to Youden Index: ≤24.4 ng/mL, sensitivity 74%, specificity 47%.

The 25(OH)D discriminative power respect the development of SARS-CoV-2 infection was explored by ROC curve analysis (Figure 2). The analysis showed an AUC of 0.61 (95%CI 0.5–0.7), *p* = 0.01. The 25(OH)D value of 24.4 ng/mL resulted the best one in terms of sensitivity, (74%) and specificity (47%).

### 3.4. Therapy

There was no significant difference between COV+ and COV− in the immunosuppressive therapy, that included prednisone in 93% of COV+ and 93% of COV-, and calcineurin inhibitors (mostly tacrolimus) in 100% of COV+ and 95% of COV−. Mycophenolate mofetil (MMF) was used in 66% and 76% of COV+ and COV−, respectively, while only 5 COV+ and 9 COV− were treated with mTor inhibitors.

iRAAS were prescribed in 26% and 16% of COV+ and COV-, respectively, with no statistically significant difference between groups (*p* = 0.14).

More than half of the studied patients had vitamin D supplementation as a part of their therapy for at least 3 months in the previous 12 months, with no significant difference between COV+ and COV− (53.3% vs. 63%, *p* = 0.27). At baseline, 101 patients (55%) had vitamin D supplementation, as calcifediol in 91% (mean daily dose: 10 ± 3 mcg) or cholecalciferol in 9% (mean daily dose: 1087 ± 984 UI). There was no difference in terms of percentage and mean daily dose of vitamin D supplementation at baseline between COV+ and COV−.

### 3.5. SARS-CoV-2 Infection Related Outcomes

Among the 61 COV+, 34 (56%) had mild symptoms and were managed at home (HOSP-), while 27 (44%) experienced a rapid evolution of respiratory symptoms with overt dyspnea and reduced oxygen saturation and were hospitalized (HOSP+). Among HOSP+, five patients (18%) were treated with non-invasive ventilation (CPAP), and two (7%) required tracheal intubation. Fifteen patients (55%) were treated with dexamethasone.

A total of six patients (10%) died due to SARS-CoV-2 infection (D+); all of them were HOSP+. 21 HOSP+ and all HOSP− survived (D−). The length of disease was higher in HOSP+ than HOSP− [25(20–36) and 20(15–27) days, respectively, *p* = 0.02].

### 3.6. Relationship between Vitamin D Status in the 12 Monts before Baseline and SARS-CoV-2 Infection Severity

Median 25(OH)D levels were: 17(8–25) ng/mL in HOSP+ and 20(15–26) ng/mL in HOSP− (*p* = 0.19); 14(6–23) ng/mL in D+ and 20(14–26) ng/mL in D− (*p* = 0.22).

Similarly, dividing the patients in HOSP−/D− (n 34, group A), HOSP+/D− (n 21, group B) and HOSP+/D+ (n 6, group C), we found progressively decreasing but not statistically different median 25(OH)D levels: group A: 20(15–26) ng/mL; group B: 19(9–26) ng/mL; group C: 14(6–24) ng/mL, *p* = 0.31.

When we considered by means of linear regression the association between 25(OH)D levels and the length of the disease, we did not find any significant correlation.

## 4. Discussion

Several studies have investigated the protective role of vitamin D in the immune response to many viral infections (such as influenza virus, respiratory syncytial virus, human immunodeficiency virus, hepatitis B and C viruses), mediated by its immunomodulatory function. As reported [10], vitamin D might enhance innate immune response, promoting antimicrobial peptides and antioxidative pathways, and lower acquired immune response, reducing T and B cell proliferation, modulating T lymphocyte cytokine production in favor of an anti-inflammatory and tolerogenic profile (inhibition of Th1 and Th17 and induction of Th2 and Tregs).

In the case of SARS-CoV-2 infection, these actions might contribute to counteract the ‘cytokine storm’ induced by the virus. Moreover, vitamin D can indirectly reduce the interaction between the virus and its intracellular entry route, namely ACE2 receptors, thanks to its negative regulation of the renin-angiotensin-aldosterone system (RAAS) [4,11,12,13,14,15,16].

Given these promises, it shouldn’t be surprising that since the SARS-CoV-2 pandemic has spread, great interest has been focused on the correlation between vitamin D status and SARS-CoV-2 infection incidence and mortality and on the effect of vitamin D supplementation on reducing the rate and severity of SARS-CoV-2 infection.

Several recent observational studies, systematic reviews, and metanalyses reported an inverse correlation between 25(OH)D levels and risk and severity of SARS-CoV-2 infection [17,18,19,20,21,22,23,24,25,26,27,28,29].

On the other hand, some RCTs have investigated the effect of vitamin D supplementation on COVID-19. Of note, Estrenas Castillo et al. [30] in a pilot randomized trial conducted among patients hospitalized for COVID-19, concluded that administration of a high dose of calcifediol in addition to best available therapy significantly reduced the need for ICU admission. However, there is still no definitive evidence that vitamin D supplementation could reduce SARS-CoV-2 infection complications [31,32].

Chronic kidney disease (CKD) and kidney transplant (KTx) are conditions associated with vitamin D deficiency and increased incidence of SARS-CoV-2 infection and mortality related to COVID-19 [5,6,7,8,33,34,35].

Most of the studies addressing the link between vitamin D status and SARS-CoV-2 infection reported a prevalence of CKD that ranged from less than 5% to 20%, while a few studies specifically focused on the CKD setting. Only two studies investigated the effect of calcitriol or paricalcitol supplementation on CKD patients with COVID-19 [36,37]. In particular, Oristrell et al. in a large observational study found that calcitriol use was associated with reduced risk of SARS-CoV-2 infection and COVID-19 severity in patients with advanced CKD (stage 4–5), probably due to their lower ability to convert 25(OH)D to 1,25(OH)2D [36].

To the best of our knowledge, this is the first study specifically addressing the association between 25(OH)D and SARS-CoV-2 infection in the setting of KTx. In order to study the influence of 25(OH)D levels on SARS-CoV-2 infection incidence and severity without confounding factors, we considered a control group of patients matched for the most important factors that could be related to SARS-CoV-2 infection risk. Moreover, differently from the majority of the studies that evaluated a single value of 25(OH)D at the time of SARS-CoV-2 infection, we considered vitamin D status as the mean of at least two 25(OH)D measurements taken during the previous year. We think that the above-mentioned characteristics can be considered the strength of present paper.

25(OH)D levels during the year before COV infection were significantly lower in COV+ than in COV-, but we did not find any difference among the other mineral metabolism parameters, renal function, urinary protein excretion, and markers of general inflammation (CRP) and nutritional status (albumin and BMI). This further supports the possible role of poor vitamin D status as a potential risk factor for SARS-CoV-2 infection. According to our opinion, the fact that the absolute difference in 25(OH)D levels between groups was small and the discriminative power of 25(OH)D was low, although statistically significant, could be due to the relatively low number of included patients.

Renal function was similar between COV+ and COV-, so we were not able to find an association between renal impairment and SARS-CoV-2 infection risk.

Although obesity is an established risk factor for SARS-CoV-2 infection [38], in our cohort we did not find any association between BMI and SARS-CoV-2 infection, possibly since most of the patients had a BMI normal to slightly overweight. Additionally, no association between the immunosuppressive therapy and the risk of SARS-CoV-2 infection was detected since the majority of our patients were treated with the same therapeutic scheme (i.e., prednisone, tacrolimus, and MMF).

Since treatment with RAASi can increase tissue expression of ACE2 and its presentation at the cell surface, concerns that treatment with RAASi might increase the risk of COVID-19 were raised. However, many subsequent studies did not find an association between RAASi use and risk of SARS-CoV-2 infection [39]. In agreement with these reports, also in our cohort we did not find any relationship between RAASi and SARS-CoV-2 infection.

With regard to vitamin D supplementation in the last year, we did not find any difference between COV+ and COV− despite differences in vitamin D status, confirming that vitamin D supplementation is frequently ineffective in restoring optimal 25(OH)D levels in the KTx setting. It important to remember, however, that vitamin D prescription is frequently characterized by a high grade of non-compliance. In routine clinical practice, our group prescribes vitamin D supplementation in the most suitable way for each patient (mostly once weekly or once a month), but we are aware that it is almost impossible to be certain of the precise compliance to vitamin D prescribed dose [40].

Another important point to underscore is that, as reported in literature, calcifediol and cholecalciferol are not equal in terms of 25(OH)D increase. Unfortunately, the design of our study and the fact that most of our patients were supplemented with calcifediol make the comparison between these formulations of vitamin D impossible [41].

Finally, we demonstrated only a trend towards a statistically significant association between 25(OH)D levels and severity of the infection. This could be highly related to the low number of events (hospitalization or death) recorded. Therefore, this correlation remains debated [42,43].

### 4.1. Limitations of the Study

We think that the main limitations of the present study are the retrospective design and the relatively small sample size, even if we report one of the highest number of kidney transplanted patients with SARS-CoV-2 infection in a single center [7]. Of course, future prospective clinical trials, obviously not desirable considering the explored topic, might better explain the potential role of 25(OH)D in modulating SARS-CoV-2 infection in this group of patients.

### 4.2. Conclusions

In conclusion, in our experience, COV+ patients showed lower 25(OH)D levels in the year preceding the infection compared to controls with similar main demographic features and comorbid conditions. No differences between groups were found in renal function parameters, proteinuria, other MM parameters, nutritional status, immunosuppressive therapy, RAASi therapy and vitamin D supplementation, and no significant association was found between 25(OH)D levels and SARS-CoV-2 infection severity.

As a bulk of evidence point at the importance of vitamin D status in protecting patients against infections and other comorbid condition after transplantation [6], we hope that more attention will be paid to the role of vitamin D in modulating infections also with prospective clinical trials.

## Figures and Tables

**Table 1:** General features of the studied patients considered globally and according to COV status (COV+ and COV−).

	Overall Cohort (*N* 183)	COV+ (*N* 61)	COV− (*N* 122)	*p*
Age, years, median (25–75%ile)	53 (46–64)	51 (44–62)	55 (46–65)	0.12
Age at KTx, years, median (25–75%ile)	44 (31–53)	42 (29–52)	45 (32–53)	0.51
KTx vintage, years, median (25–75%ile)	8 (3–15)	8 (3–15)	7 (2–15)	0.78
Gender, *n* (F/M, %)	72/111 (39/61)	24/37 (39/61)	48/74 (39/61)	0.87
Diabetes, *n* (%)	33 (18)	11 (18)	22 (18)	0.81
Hypertension, *n* (%)	156 (85)	52 (85)	104 (85)	0.82
Symptomatic heart disease, *n* (%)	6 (3)	2 (3)	4 (3)	0.66

Footnotes: KTx, kidney transplantation; F, females; M, males.

**Table 2:** Differences in vitamin D status and other evaluated anthropometric and biochemical parameters between COV+ and COV− in the 12 months before baseline.

	COV+ (N 61)	COV− (N 122)	*p*
BMI, kg/m^2^	24.5 (21.7–26.9)	24.2 (21.1–26.7)	0.99
25(OH)D, ng/mL	19 (12–26)	23 (17–31)	0.01
1,25(OH)_2_D, pg/mL *	42 (34–49)	48 (34–58)	0.14
Ca, mg/dL	9.5 (9.1–9.9)	9.4 (9.2–9.8)	0.73
P, mg/dL	3.1 (2.6–3.5)	2.9 (2.6–3.4)	0.44
Mg, mg/dL	1.8 (1.6–1.8)	1.7 (1.6–1.8)	0.09
PTH, pg/ml	59 (41–85)	61 (46–91)	0.37
Serum Creatinine, mg/dL	1.4 (1.2–1.8)	1.3 (1.1–1.8)	0.09
U-Prot, gr/day	0.15 (0.11–0.33)	0.18 (0.13–0.31)	0.53
Serum Albumin, mg/dL	4.2 (3.9–4.4)	4.1 (3.9–4.3)	0.46
CRP, mg/dL	0.2 (0–1–0.4)	0.2 (0.1–0.5)	0.56

Footnotes: BMI, body mass index; 25(OH)D, 25-hydroxyvitamin D; 1,25(OH)2D, 1,25-dihydroxyvitamin D; PTH, Parathormone; U-Prot, daily urinary protein excretion; CRP, C-reactive protein; * data available in 44 COV+ and 88 COV−.

## Data Availability

The data presented in this study are available on request from the corresponding author. The data are not publicly available due to privacy issues.
